# Structurally Tunable Reduced Graphene Oxide Substrate Maintains Mouse Embryonic Stem Cell Pluripotency

**DOI:** 10.1002/advs.201802136

**Published:** 2019-04-17

**Authors:** Jinping Zhao, Mingliang Tang, Jing Cao, Dan Ye, Xudong Guo, Jiajie Xi, Yi Zhou, Yuchen Xia, Jing Qiao, Renjie Chai, Xiaowei Yang, Jiuhong Kang

**Affiliations:** ^1^ Clinical and Translational Research Center of Shanghai First Maternity and Infant Health Hospital School of Life Science and Technology Tongji University Shanghai 200092 China; ^2^ Institute for Regenerative Medicine Shanghai East Hospital School of Materials Science and Engineering Tongji University Shanghai 200092 China; ^3^ Key Laboratory for Developmental Genes and Human Disease Ministry of Education Institute of Life Sciences Jiangsu Province High‐Tech Key Laboratory for Bio‐Medical Research Southeast University Nanjing 210096 China; ^4^ Co‐Innovation Center of Neuroregeneration Nantong University Nantong 226001 China; ^5^ Institute for Stem Cell and Regeneration Chinese Academy of Science Beijing 100864 China

**Keywords:** E‐cadherin, embryonic stem cells, pluripotency, reduced graphene oxide, Wnt signaling pathway

## Abstract

Culturing embryonic stem cells (ESCs) in vitro usually requires animal‐derived trophoblast cells, which may cause pathogenic and immune reactions; moreover, the poor repeatability between batches hinders the clinical application of ESCs. Therefore, it is essential to synthesize a xenogeneic‐free and chemically well‐defined biomaterial substrate for maintaining ESC pluripotency. Herein, the effects of structurally tunable reduced graphene oxide (RGO) substrates with different physicochemical properties on ESC pluripotency are studied. Colony formation and CCK‐8 assays show that the RGO substrate with an average 30 µm pore size promotes cell survival and proliferation. The unannealed RGO substrate promotes ESC proliferation significantly better than the annealed substrate due to the interfacial hydrophilic groups. The RGO substrate can also maintain ESC for a long time. Additionally, immunofluorescence staining shows that ESCs cultured on an RGO substrate highly express E‐cadherin and β‐catenin, whereas after being modified by Dickkopf‐related protein 1, the RGO substrate is unable to sustain ESC pluripotency. Furthermore, the cell line that interferes with E‐cadherin is also unable to maintain pluripotency. These results confirm that the RGO substrate maintains ESC pluripotency by promoting E‐cadherin‐mediated cell–cell interaction and Wnt signaling.

## 1 Introduction

Embryonic stem cells (ESCs), derived from the inner cell mass of the blastula stage, are pluripotent stem cells with high self‐renewal capacity and can differentiate into all cell types of the three germ layers,^1^, ^2^, ^3^ thus making them an ideal material for disease models, basic biomedical research, and regenerative medicine.^4^, ^5^, ^6^, ^7^ Since isolated ESCs often undergo uncontrolled differentiation in vitro, maintaining the pluripotency of ESCs in vitro is challenging.^8^ Currently, the general practice of culturing ESCs in vitro relies on trophoblast cells (feeder),^9^ with some researchers also choosing animal‐derived matrigel or an extracellular matrix (ECM). However, the above methods, including feeder, matrigel, and ECM, share several disadvantages: first, they may carry pathogens and immunogens, which tend to cause diseases and immune reactions of ESCs; second, their compositions are biologically complex and cannot be clearly defined; finally, their quality may vary greatly between batches. Moreover, matrigel and ECM generally require the conditioned medium to maintain the pluripotency of ESCs.^10^, ^11^ Therefore, xenogeneic‐free and chemically well‐defined synthetic substrates provide unique advantages in culturing ESCs. For example, mouse ESCs are able to maintain self‐renewal capacity on 2‐hydroxyethyl methacrylate‐*co*‐ethylene dimethacrylate substrate with a micro–nano rough surface for a prolonged time, which is achieved by reducing the integrin signal of mouse ESCs.^12^ Human ESCs maintain pluripotency on polystyrene nanopillar substrates with a diameter of 120–170 nm, mainly due to cell–nanotopography interactions that regulate focal adhesion formation and cytoskeleton reorganization, and enhance E‐cadherin‐mediated cell–cell adhesion in clones.^13^ It has also been demonstrated that the physicochemical properties of the substrate, such as surface roughness, pattern, and chemical group, could affect the cell behavior and pluripotency of ESCs.^14^, ^15^, ^16^, ^17^ However, the substrates mentioned above have common disadvantages, such as they have poor chemical properties, are unable to arbitrarily adjust the content of oxygen‐containing groups, are difficult to be modified by proteins, cannot be mass‐produced, exhibit changes in properties after folding or bending, and cannot be transplanted into the body as a scaffold.

Graphene and its derivatives have unique physicochemical properties, such as easy surface modification, excellent electrical conductivity, high chemical stability, good flexibility, high strength, and so on. They have been widely used in the research of stem cells.^18^, ^19^, ^20^, ^21^ For example, graphene could promote the differentiation of embryoid bodies into cardiomyocytes, which is more pronounced after applying electrical stimulation.^22^ The nGO@Fe_3_O_4_ magnetic particles possessing numerous bindable carboxyl groups could carry bone morphogenetic protein 2 (BMP 2) and transforming growth factor β 3 (TGFβ 3) after being modified by graphene and related derivatives; therefore, the composite particles could regulate the differentiation of stem cells after being transported into cells.^23^ However, studies on whether graphene and its derivatives can maintain the pluripotency of ESCs in the absence of a feeder in vitro have not been widely reported. At present, the suspended graphene oxide (GO) nanosheets in the culture medium have been reported to promote and maintain the self‐renewal ability of ESCs,^24^ proving the good biocompatibility of GOs; however, suspended GOs are nonportable, nonconductive, and cannot be used as a scaffold for the subsequent clinical application. Therefore, it is critical to design and synthesize a free‐standing, easily modifiable, electrically conductive, and chemically stable graphene substrate that can maintain ESC pluripotency in vitro instead of with feeders.

In both biological and synthetic scaffolding culture systems, the pluripotency of ESCs is mostly controlled by intracellular factor regulatory networks and exogenous signal pathways.^25^ The ECM, which serves as a carrier for signal transmission between cells, can interact with cell surface receptors to transmit intracellular signals. The ECM and the extracellular domain of cadherin, a type of transmembrane glycoprotein, play an important role in cell adhesion. E‐cadherin, a member of the cadherin family, enables ESC clones to be more compact by promoting cell–cell interactions;^14^ the recombinant E‐cadherin also promotes the sustained pluripotency of ESCs.^26^ The cytoplasmic domains of E‐cadherin and β‐catenin are closely related in mediating cell adhesion and stem maintenance. For example, these substrates can maintain the pluripotency of pluripotent stem cells by regulating the expression of E‐cadherin and β‐catenin.^13^, ^14^, ^16^ 3D elastin‐like protein hydrogels are able to maintain neural progenitor cell (NPC) stemness via the modulation of cadherin‐mediated β‐catenin signaling.^27^ However, with regard to graphene substrates and related derivatives, whether the substrate is able to maintain the pluripotency of ESCs by promoting E‐cadherin‐mediated β‐catenin signaling remains unclear.

Herein, we have designed and synthesized reduced graphene oxide (RGO) substrates, aiming to study the effects of RGO substrates with different pore sizes and oxygen‐containing group content on ESC behavior and their fate in the absence of feeder in vitro; associated mechanisms are also explored. We found that ESCs on the unannealed RGO substrate, with an average 30 µm pore size, not only had the strongest adhesion and proliferation abilities, but also maintained the pluripotency for a long time without sacrificing the self‐renewal ability and multilineage potential. After further experimental verification, we demonstrate for the first time that RGO substrates maintain ESC pluripotency by regulating the E‐cadherin/Wnt signaling pathway.

## 2 Results

### 2.1 Fabrication and Characterization of RGO Substrates

**Figure 1:**
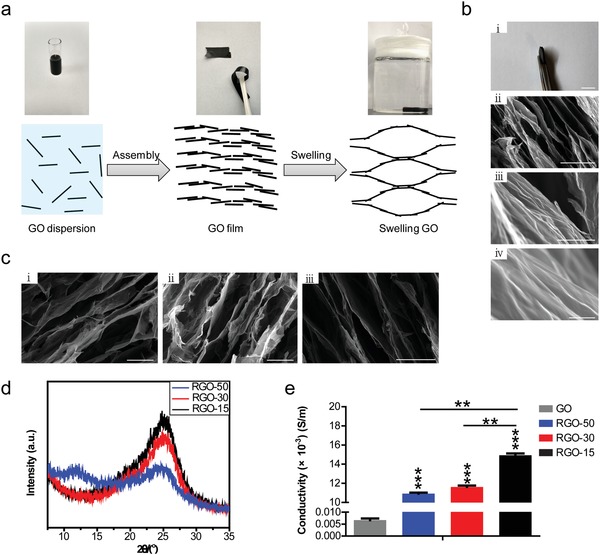
Fabrication and characterization of the RGO substrate. a) Schematic diagram of the synthetic preparation of RGO substrates. b‐i) A macroscopic picture of the substrate obtained from panel (a); ii–iv)SEM images of the RGO substrates with different magnifications (ii: 1000 ×; iii: 15 000 ×; iv: 60 000 ×). Scale bars: 1 cm in (i), 40 µm in (ii), 3 µm in (iii) and 500 nm in (iv). c) The RGO substrates with pore sizes of 50 µm (i), 30 µm (ii) and 15 µm (iii) were confirmed by SEM. Scale bars: 50 µm in (i,ii) and 30 µm in (iii). d) The X‐ray diffraction pattern analysis of RGO‐50, RGO‐30, and RGO‐15 substrates. e) The conductivities of RGO‐50, RGO‐30, and RGO‐15 substrates, and GO film used as a control. ** *p* < 0.01 and *** *p* < 0.001.

The synthesis of the RGO substrates is shown by the schematic diagram (**Figure** **1**a). Briefly speaking, the obtained GO dispersion was converted into GO film after being dried. The GO films, which could be punched into arbitrary sizes, were immersed in l‐ascorbic acid solution. The ions in the solution interacted with the oxygen‐containing groups of the GO layers to form cross‐links, which generally attained a balance with the repulsive interactions among the GO layers. Simultaneously, the GO was reduced to RGO by l‐ascorbic acid. Finally, the anisotropic porous RGO substrate was obtained by freeze‐drying.

The scanning electron microscope (SEM) images exhibit an anisotropic cellular pore structure that was assembled by graphene sheets, and revealed that all the graphene sheets are aligned and interconnected from each other to form controllable micropores (Figure 1b). It could be seen from the high‐magnification SEM images that there are many wrinkles on the walls of the RGO substrate (Figure 1b), which is favorable for cell adhesion. In this study, we obtained three substrates with different pore sizes: 50, 30, and 15 µm (RGO‐50, RGO‐30, and RGO‐15), which were also verified by SEM (Figure 1c). The X‐ray diffraction (XRD) patterns of these RGO substrates with different pore sizes are shown in Figure 1d. The RGO‐50, RGO‐30, and RGO‐15 substrates all show the diffraction peaks at 2θ = 25.2° with varying intensities, indicating that most of the GO in the three substrates has been reduced. Following an increase in pore size, the intensity of the peak becomes weaker (Figure 1d), possibly due to an increase in the extent of disorder. The RGO‐50 substrate exhibits a weak and wide diffraction peak at 2θ = 12° (Figure 1d), showing GO is still present. As shown in Figure 1e, the conductivities of the RGO substrates with different pore sizes maintain a range of 10–15 × 10^−3^ S m^−1^ (the sheet resistances at thousands Euclidean). The conductivity of RGO‐15 is significantly higher than that of RGO‐30, both of which are significantly higher than RGO‐50 (Figure 1e). The conductivities of the three RGO substrates are significantly higher than that of GO film (Figure 1e), proving that they are reduced.

### 2.2 The Self‐Renewal Ability of ESCs Cultured on RGO Substrates with Different Pore Sizes

**Figure 2:**
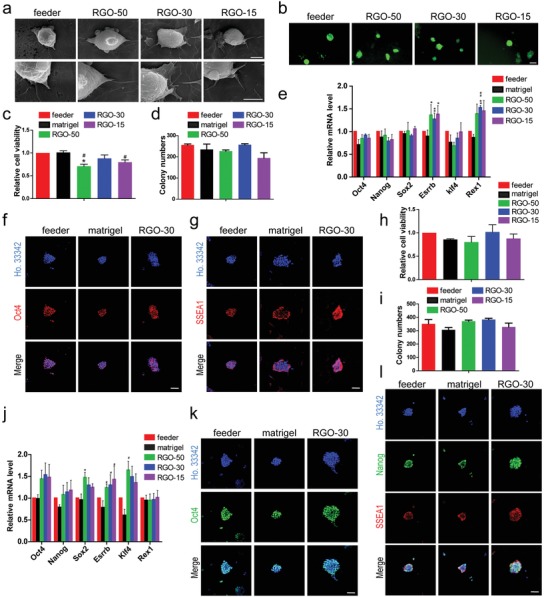
RGO substrate phenocopy feeder cells to support ESC self‐renewal. a) The SEM images with different magnifications of a single cell of NR3 cultured on feeder or RGO substrates for 4 h. Magnifications: 5000 × (the upper line) and 10 000 × (the lower line). Scale bars: 5 µm in the upper line and 4 µm in the lower line. b) Nanog‐green fluorescent protein expressing ESCs (NR3) were cultured on feeder, RGO‐50, RGO‐30, and RGO‐15 substrates for 2 days, respectively. The pictures were taken by a fluorescence microscope. Scale bar: 100 µm. c) The relative cell viability of NR3 on feeder, matrigel, RGO‐50, RGO‐30, and RGO‐15 substrates. ^#^ *p* < 0.05 versus matrigel group and * *p* < 0.05 versus feeder group. d) The alkaline phosphatase staining of NR3 cultured on feeder, matrigel, and RGO substrates was performed, and the number of positive clones was statistically analyzed. e) The expression levels of pluripotency gene Oct4, Nanog, Sox2, Esrrb, Klf4, and Rex1 of NR3 cultured on feeder, matrigel, and RGO substrates were detected by qRT‐PCR. ^##^ *p* < 0.01 versus matrigel group and * *p* < 0.05, ** *p* < 0.01 versus feeder group. The immunofluorescent staining of f) Oct4 and g) SSEA1 of NR3 cultured on feeder, matrigel, and RGO‐30 substrates. Oct4 and SSEA1 (stage‐specific embryonic antigen 1) are pluripotency marker proteins of mouse ESCs. Scale bar: 50 µm in panels (f) and (g). h) The relative cell viability of 46C on feeder, matrigel, and RGO substrates. i) Alkaline phosphatase assay of 46C cultured on feeder, matrigel, RGO‐50, RGO‐30, and RGO‐15 substrates, and the number of positive clones was counted. j) The expression levels of Oct4, Nanog, Sox2, Esrrb, Klf4, and Rex1 of 46C cultured on feeder, matrigel, and RGO substrates. ^#^ *p* < 0.05 versus matrigel group and * *p* < 0.05 versus feeder group. The protein levels of k) Oct4, l) Nanog, and SSEA1 expressed by 46C on feeder, matrigel, and RGO‐30 substrates were evaluated by immunofluorescent staining. Scale bar: 50 µm in panels (k) and (l).

We first investigated the effect of RGO substrates with different pore sizes on the pluripotency of ESCs. In order to intuitively explore the interaction between ESCs and substrate, Nanog‐green fluorescent protein expressing mouse ESC line NR3 was selected to monitor the expression pattern of Nanog. The NR3 is inoculated onto RGO‐50, RGO‐30, and RGO‐15 substrates as well as on feeder. In the single‐cell state, the cells on the feeder and three RGO substrates could quickly extend filopodia anchoring to the substrate (**Figure** **2**a), indicating the good biocompatibility of RGO substrates.

After proliferation, NR3 on the feeder and three RGO substrates retain in colonies with the green fluorescence (Figure 2b). In order to detect the effects of RGO substrate on ESC survival and self‐renewal, we conducted Cell Counting Kit‐8 (CCK‐8) and colony formation assays, and took matrigel as the feeder‐free control. Although the relative cell viability of the feeder and matrigel groups are higher than those of the RGO‐50 and RGO‐15 substrates, there is no significant difference from the RGO‐30 substrate (Figure 2c). The clones with positive alkaline phosphatase (AP) on feeder, matrigel, RGO‐50, RGO‐30, and RGO‐15 substrates are 256, 235, 227, 257, and 194, respectively (Figure 2d).

The NR3 was collected separately, and the expression levels of the pluripotency genes were detected by quantitative real‐time polymerase chain reaction (qRT‐PCR). Although the levels of Oct4, Nanog, Sox2, and Klf4 expressed by NR3 on RGO‐50, RGO‐30, and RGO‐15 substrates are slightly lower than that of the feeder group, the levels are not much different from that of matrigel group (Figure 2e). The expression levels of Esrrb and Rex1 of NR3 on the three RGO substrates are higher than that on the feeder and matrigel (Figure 2e). It is worth noting that compared with RGO‐50 and RGO‐15 substrates, RGO‐30 substrate has the best effect on cell viability and self‐renewal, and significantly promotes ESC expression of pluripotency protein. Therefore, we chose RGO‐30 for the subsequent experiments. For further validation, we examined the expression levels of the pluripotency proteins of NR3 cultured on each substrate. It is found by immunofluorescence staining that, like the feeder and matrigel groups, the RGO‐50, RGO‐30, and RGO‐15 substrates promote NR3 highly expressing Oct4 and stage‐specific embryonic antigen 1 (SSEA1) (Figure 2f,g; Figure S1a,b,Supporting Information).

To test whether the property of the RGO substrates maintaining NR3 pluripotency is applicable to other mouse ESC lines, we employed another commonly used cell line—46C. Like the matrigel group, the cell viability of RGO‐50 and RGO‐15 substrates is slightly lower than that of the feeder and RGO‐30 groups (Figure 2h). For the AP staining, the AP‐positive clones of 46C on feeder, matrigel, RGO‐50, RGO‐30, and RGO‐15 substrates are351, 306, 371, 383, and 328, respectively (Figure 2i). The expression levels of the pluripotency genes of 46C on three RGO substrates are higher than that of the matrigel group, some being even higher than that of the feeder group (Figure 2j). Since Nanog carried by NR3 is one of the most important markers for mouse ESCs, we examined the protein expression level of Nanog of 46C on each substrate. Consistent with NR3, immunofluorescence staining shows that the positive fluorescence signals of 46C on the three RGO substrates are very strong (Figure 2k,l; Figure S1c,d, Supporting Information).

All these results demonstrate that like the feeder and matrigel, three RGO substrates can promote the ESCs expressing pluripotency genes and proteins, among which the RGO‐30 substrate promotes ESC survival and self‐renewal most obviously.

### 2.3 The Multilineage Differentiation Potential of ESCs Grown on RGO Substrates

**Figure 3:**
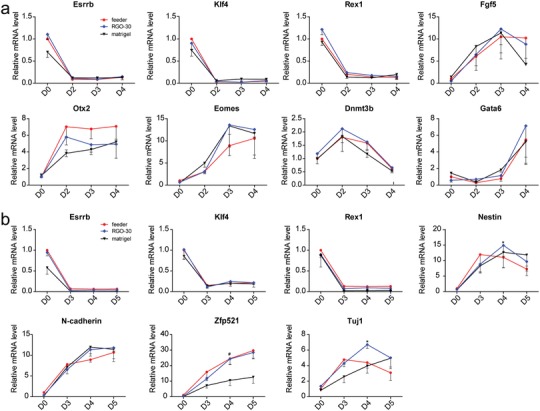
ESCs culturing on RGO substrates possess multilineage differentiation potential. a) The relative levels of pluripotency gene (Esrrb, Klf4, and Rex1) and mesendoderm gene (Fgf5, Otx2, Eomes, Dnmt3b, and Gata6) during early differentiation expressed by 46C cultured on feeder, matrigel, and RGO‐30 substrates. b) The relative expression levels of Esrrb, Klf4, Rex1, Nestin, N‐cad (N‐cadherin), Zfp521 (zinc finger protein 521), and Tuj1 in 46C cultured on feeder, matrigel, and RGO‐30 substrates were assessed by qRT‐PCR. Nestin, N‐cad, and Zfp521 are markers of ectoderm, and Tuj1 is a marker of neuron. ^#^ *p* < 0.05 versus matrigel group.

Since we proved that the properties of RGO substrate promoting ESCs expressing pluripotency proteins and sustaining self‐renewal ability are universal for mouse ESCs, in order to avoid the waste of resources, 46C was selected for the follow‐up experiments. The spontaneous differentiation of ESCs cultured on feeder, matrigel, and RGO substrates was performed. The pluripotency genes and early differentiation genes of mesendoderm were detected by qRT‐PCR. At the beginning of differentiation, the expression levels of the pluripotency genes are rapidly downregulated, and the levels of early differentiation genes of mesendoderm begin to upregulate (**Figure** **3**a; Figure S2a, Supporting Information). There is no significant difference in differentiation process and efficiency. Since the spontaneous differentiation of ESCs is mainly toward mesendoderm, in order to comprehensively test the differentiation potential of ESCs cultured on the RGO substrates, we added retinoic acid (RA) to the differentiation medium to induce ESCs to differentiate into ectoderm. The pluripotency genes and early ectoderm genes were also detected by qRT‐PCR. Similar to the spontaneous differentiation, the expression levels of the pluripotency genes downregulate immediately after differentiation, and the levels of early ectodermal genes begin to upregulate (Figure 3b; Figure S2b, Supporting Information). It was found by spontaneous and directional differentiation that ESCs cultured on RGO substrates possess multiple differentiation potentials.

In the previous experiments of this study, we demonstrate that the RGO substrates not only possess good biocompatibility, but also promote ESCs expressing pluripotency genes and proteins. In particular, the RGO‐30 substrate facilitates the self‐renewal ability of ESCs. Now, we have found that ESCs cultured on RGO substrates possess multilineage differentiation potential. It can be concluded that the RGO substrates designed and synthesized in this study promote ESCs, maintaining pluripotency, in the absence of feeder.

### 2.4 The Effects of Different Oxygen‐Containing Group Content of the RGOs on the Behavior of ESCs

As already mentioned, the RGO‐30 substrate is better than RGO‐50 and RGO‐15 in promoting cell survival and self‐renewal (Figure 2c,d,h,i). Therefore, RGO‐30 substrate was chosen for subsequent experiments. Based on the study of the effects of different physical properties on the maintenance of ESC pluripotency, we planned to study the effects of different chemical properties on ESC fate. Since the content of oxygen‐containing groups of the RGO substrates directly affects the protein‐binding capacity, hydrophilicity, cell adhesion, etc.,^28^, ^29^, ^30^ we selected RGO substrates with different degrees of reduction (content of oxygen‐containing groups) to detect the effects on cell behavior and fate. Therefore, we annealed RGO‐30 substrates at room temperature, 300 and 600 °C, and obtained RGO substrates with different oxygen‐containing group content, expressed as RGO‐RT, RGO‐300, and RGO‐600, respectively.

**Figure 4:**
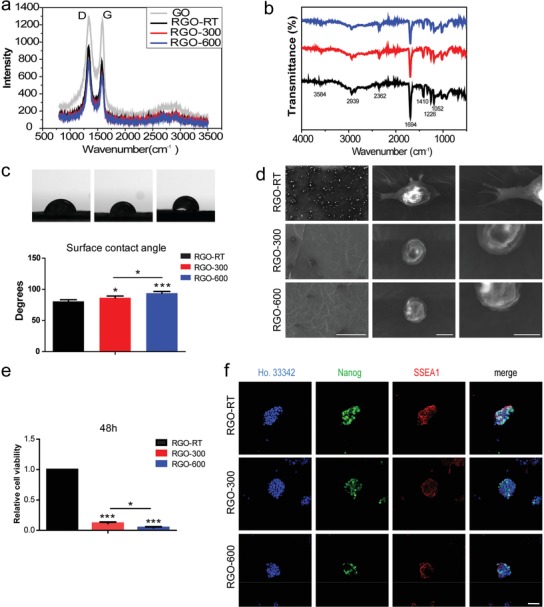
Oxygen‐containing groups are critical for RGO substrate to promote ESC adhesion and proliferation. The RGO‐30 substrates were annealed at room temperature, 300 and 600 °C, and are expressed as RGO‐RT, RGO‐300, and RGO‐600, respectively. a) Raman spectra of RGO‐RT, RGO‐300, and RGO‐600 substrates. b) The oxygen‐containing group content of the RGO‐RT, RGO‐300, and RGO‐600 substrates was analyzed by FTIR. c) The hydrophilicity of RGO‐RT, RGO‐300, and RGO‐600 substrates was detected by surface contact angle measurements. * *p* < 0.05, *** *p* < 0.001. d) SEM images with different magnifications of a single cell of 46C cultured on RGO‐RT, RGO‐300, or RGO‐600 substrate for 4 h. Magnifications: 150 × (the left column), 5000 × (the middle column), and 10 000 × (the right column). Scale bars: 300 µm in the left column, 5 µm in the middle column, and 4 µm in the right column. e) The relative cell viability of 46C cultured on RGO‐RT, RGO‐300, and RGO‐600 substrates for 48 h was detected by CCK‐8 kit. * *p* < 0.05, *** *p* < 0.001. f) The protein expression levels of Nanog and SSEA1 were assessed by immunofluorescent staining. Scale bar: 50 µm.

It could be seen from Raman spectra that the D band of GO is significantly higher than that of RGO‐RT, RGO‐300, and RGO‐600 substrates; the G band gradually weakens then enhances following an increase in the anneal temperature (**Figure** **4**a), confirming that the RGOs are further reduced and the defects on the GO layers simultaneously decrease. Additionally, Fourier transform infrared spectroscopy (FTIR) was used to analyze the oxygen‐containing group content of the substrates. The peak values at 1052, 1226, 1410, and 1694 cm^−1^ correspond to carbon–oxygen single bonds, epoxy bonds, hydroxyl groups and carbon–oxygen double bonds, respectively. Following an increase in the anneal temperature, the peak intensities are weakened (Figure 4b), indicating that the content of oxygen‐containing groups (hydroxyl group, carboxyl group, and epoxy group, etc.) decreases with an increasing degree of reduction. At the same time, the contact angles of the substrate gradually increase significantly (Figure 4c), proving the gradual decrease of the hydrophilicity of the substrate.

We observed single cells cultured on RGO‐RT, RGO‐300, and RGO‐600 substrates by SEM. At low magnification, there are many cells adhering to the RGO‐RT substrate, compared with the significantly low cell numbers on RGO‐300 and RGO‐600 substrates (Figure 4d). It could also be seen that the cells on the RGO‐RT substrate stretch out filopodia to anchor to the substrate, whereas a few filopodia protrude from the cells on the RGO‐300 and RGO‐600 substrates (Figure 4d), proving that the RGO‐RT substrate is more conducive to cell adhesion. After 48 h of proliferation culture, the amount of ESCs on each substrate was measured by CCK‐8 assay. The cell numbers of RGO‐300 and RGO‐600 groups are only reached 11.7% and 4.6% of the RGO‐RT group, respectively (Figure 4e), indicating that as the number of oxygen‐containing groups decreases, the number of cells reduces. Thus, the unannealed RGO‐RT substrate synthesized in this study could promote ESC proliferation, whereas the RGO‐300 and RGO‐600 substrates are not conducive to the adhesion and proliferation of ESCs. The conductivity of RGO‐RT is significantly higher than that of GO film (Figure 1e); therefore, RGO‐RT balances the characteristics of electrical conductivity and oxygen‐containing groups well.

Immunofluorescence staining was performed on ESCs cultured on RGO‐RT, RGO‐300, and RGO‐600 substrates to detect the expression level of the pluripotency protein. Although partial cells on RGO‐300 and RGO‐600 substrates are Nanog positive and SSEA1 positive, the fluorescence intensities of the clones are weaker than that of the RGO‐RT group, and the percentages of positive cells on the RGO‐300 and RGO‐600 substrates are also significantly reduced (Figure 4f); this further proves that the chemical properties of the RGO‐RT substrate are more suitable for the adhesion and proliferation of ESCs than the RGO‐300 and RGO‐600 substrates.

### 2.5 Long‐Term Cultivation of ESC on RGO Substrate

**Figure 5:**
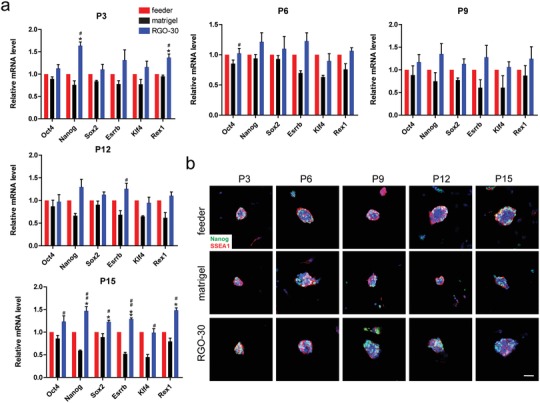
Long‐term cultivation of ESC on RGO substrate. a) qRT‐PCR was used to detect the expression level of pluripotency gene after continuous subculture of 46C on feeder, matrigel, and RGO‐30 substrates. ^#^ *p* < 0.05, ^##^ *p* < 0.01 versus matrigel group, and * *p* < 0.05, ** *p* < 0.01 versus feeder group. b) The expression levels of Nanog(green) and SSEA1(red) in 46C after continuous subculture was tested by immunofluorescent staining. P3/6/9/12/15 stand for passage 3/6/9/12/15. Scale bar: 50 µm.

In order to test whether the RGO substrate could maintain ESC for a long time, we continuously subcultured the ESCs on RGO substrate, and collected cells with different passages for qRT‐PCR and immunofluorescence staining. Because of time constraints, we tested the pluripotency of ESCs within 30 days (15 passages). It was found that during the test period, RGO substrate promoted ESC expression of pluripotency genes and proteins, maintaining the pluripotency of ESCs (**Figure** **5**a,b).

### 2.6 The Effects of RGO Substrate on Human ESCs

**Figure 6:**
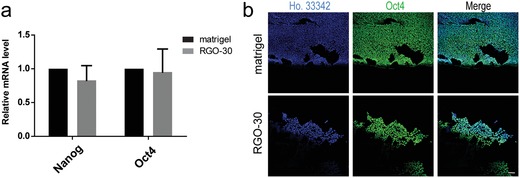
The effects of RGO substrate on human ESCs. a) The expression levels of pluripotency gene in human ESC on matrigel and RGO‐30 substrate were detected by qRT‐PCR. b) Immunofluorescent staining revealed the protein expression level of Oct4 in human ESC on matrigel and RGO‐30 substrate. Scale bar: 100 µm.

In addition, human ESC was also inoculated on the RGO substrate. qRT‐PCR analysis showed that although the expression level of pluripotency gene in human ESC cultured on RGO substrate was lower than that of matrigel group, there was no significant difference between the two (**Figure** **6**a). It was found by immunofluorescence staining that human ESC on RGO substrate could express pluripotency protein, which was similar to the situation of mouse ESC (Figure 6b). The number of cells on RGO substrate is less than that on matrigel, possibly because the RGO is being coated by gelatin only, and matrigel is more conducive to ESC adhesion than gelatin.

### 2.7 E‐Cadherin/β‐Catenin Are Activated in ESCs Cultured on RGO Substrate

**Figure 7:**
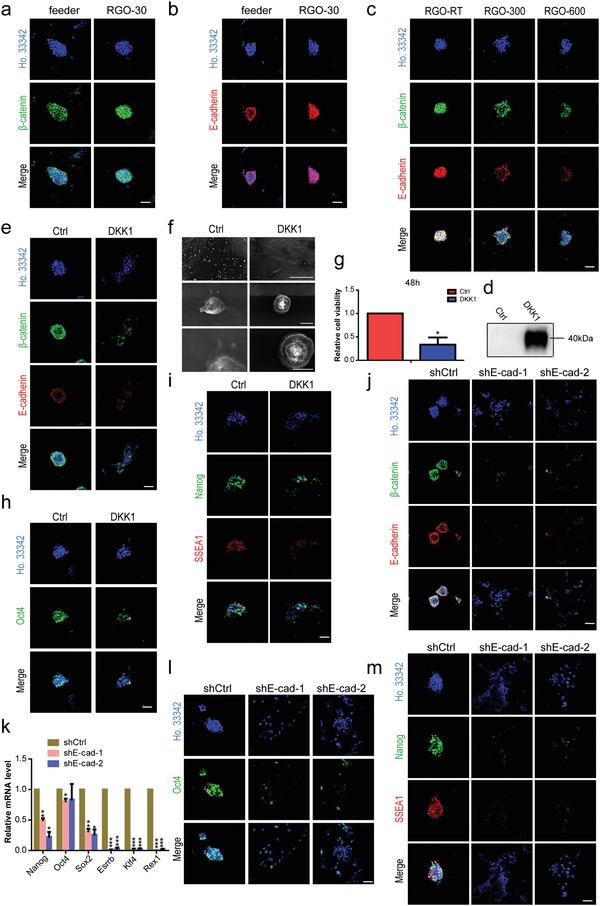
RGO substrates activate β‐catenin/E‐cadherin expression in maintaining ESC pluripotency. The immunofluorescent staining of a) pluripotency‐maintaining protein β‐catenin and b) cell adhesion–associated protein E‐cadherin of 46C cultured on feeder and RGO‐30 substrates. Scale bar: 50 µm in panels (a) and (b). c) The protein expression levels of β‐catenin (green) and E‐cadherin (red) in 46C cultured on RGO‐RT, RGO‐300, and RGO‐600 substrates. Scale bar: 50 µm. d) Western blotting of RGO‐30 substrates after being modified by water and DKK1, respectively. Dickkopf related protein 1 (DKK1) was the inhibitor of the classic Wnt signaling pathway. Water‐modified substrate was used as a control (Ctrl). e) The protein expression levels of β‐catenin (green) and E‐cadherin (red) in 46C cultured on water or DKK1‐modified substrate. Scale bar: 50 µm. f) The SEM images with different magnifications of a single cell of 46C cultured on water or DKK1‐modified substrate for 4 h. Magnifications: 150 × (the upper line), 5000 × (the middle line), and 10 000 × (the lower line). Scale bars: 300 µm in the upper line, 5 µm in the middle line, and 4 µm in the lower line. g) The relative cell viability of 46C cultured on water or DKK1‐modified substrate for 48 h was detected by CCK‐8 kit. * *p* < 0.05 versus water‐modified substrate. The protein expression levels of h) Oct4, i) Nanog and SSEA1 in 46C cultured on water or DKK1‐modified substrate were detected by immunofluorescent staining. Scale bar: 50 µm in panels (h) and (i). The protein levels of j) β‐catenin and E‐cadherin of shCtrl, shE‐cad‐1, and shE‐cad‐2 on RGO‐30 substrate. Scale bar: 50 µm. k) The expression levels of Nanog, Oct4, Sox2, Esrrb, Klf4, and Rex1 of shCtrl, shE‐cad‐1, and shE‐cad‐2 cultured on RGO‐30 substrate were detected by qRT‐PCR. * *p* < 0.05, ** *p* < 0.01, *** *p* < 0.001 versus the shCtrl group. The representative fluorescence microscope images of l) Oct4, m) Nanog, and SSEA1 expressed by shCtrl, shE‐cad‐1, and shE‐cad‐2 on RGO‐30 substrate. Scale bar: 50 µm.

It has been previously reported that the substrate could regulate cell behavior and fate by regulating the expression of β‐catenin and E‐cadherin.^13^, ^14^, ^16^ Here, we examine the protein expression levels of β‐catenin and E‐cadherin in ESCs and find that the expression levels of β‐catenin and E‐cadherin in ESCs cultured on RGO‐50, RGO‐30, and RGO‐15 substrates are similar to that of the feeder group (**Figure** **7**a,b; Figure S3a–d, Supporting Information). This phenomenon occurs in both 46C and NR3, implying that the RGO substrate works in the same manner in both cell lines. Although ESCs cultured on RGO‐300 and RGO‐600 substrates could express β‐catenin and E‐cadherin, the percentages of positive cells are less than the RGO‐RT group (Figure 7c). ESCs cultured on RGO‐50, RGO‐30, and RGO‐15 substrates could highly express β‐catenin and E‐cadherin, while β‐catenin is not only closely related to the cytoplasmic region of E‐cadherin in mediating cell adhesion, but also a transcription factor of the classic Wnt signaling pathway that regulates mouse ESC pluripotency. Therefore, we hypothesize that the RGO substrate maintains mouse ESC pluripotency, possibly by activating the E‐cadherin/Wnt signaling pathway.

To verify the above assumption, we designed two experimental programs. On one hand, since the RGO substrates contain a large number of oxygen‐containing groups and the protein can interact with carboxyl groups, we modified the RGO‐30 substrate with Dickkopf‐related protein 1 (DKK1), an inhibitor of the classic Wnt signaling pathway. It was confirmed by western blotting that the RGO‐30 substrate was successfully modified by DKK1 (Figure 7d). ESCs cultured on DKK1‐modified substrate not only downregulate the expression level of β‐catenin, but also inhibit the expression of E‐cadherin (Figure 7e), suggesting that the cell–cell interactions are impeded. SEM images show that compared with the control group, the cells on DKK1‐modified substrate rarely extend filopodia (Figure 7f), consistent with the protein expression level of E‐cadherin. The number of cells was detected by CCK‐8 after 48 h of culture proliferation, showing that the number of cells on DKK1‐modified substrate is only 33.57% of the control group (Figure 7g). At the same time, the expression levels of Oct4, Nanog, and SSEA1 of ESCs on the DKK1‐modified substrate are lower than that of the control group (Figure 7h,i), proving that the RGO substrate maintains ESC pluripotency mostly by promoting the classic Wnt signaling pathway. Additionally, we constructed cell lines (shE‐cad‐1, shE‐cad‐2) that interfered with E‐cadherin, which was confirmed by western blotting, qRT‐PCR, and immunofluorescence staining (Figure 7j; Figure S3e,f, Supporting Information). The expression levels of β‐catenin in shE‐cad‐1 and shE‐cad‐2 are downregulated (Figure 7j). It can thus be concluded that not only are the gene expression levels of Oct4, Nanog, Sox2, Klf4, Esrrb, and Rex1 in shE‐cad‐1 and shE‐cad‐2 significantly lower than those of the shCtrl group (Figure 7k), but fluorescence intensities of Nanog, SSEA1, and Oct4 in shE‐cad‐1 and shE‐cad‐2 are also weaker than those of the shCtrl group (Figure 7l,m). The proportions of positive cells of shE‐cad‐1 and shE‐cad‐2 are significantly reduced, and shE‐cad‐1 and shE‐cad‐2 are unable to maintain a conal shape (Figure 7l,m). These results demonstrate that maintaining the pluripotency of ESCs on RGO substrates is dependent on E‐cadherin. Combining the results of the two experimental programs, the RGO substrate can be seen to maintain ESC pluripotency by activating the E‐cadherin/Wnt signaling pathway.

## 3 Discussion

In this study, RGO substrates could promote the survival and proliferation of ESCs, without sacrificing pluripotency, based on the good biocompatibility of graphene and its derivatives.[qv: 19,21,]^31^, ^32^ Many scholars have reported that the physical properties of the substrates can regulate the pluripotency of ESCs.^12^, ^13^, ^14^, ^15^ In our studies, the RGO‐30 substrate shows better promotion for the self‐renewal of ESCs compared with RGO‐50 and RGO‐15 substrates, consistent with the conclusions of other scholars.^12^, ^13^, ^14^, ^15^ Compared with traditional feeder culture systems, RGO substrates possess many advantages with regard to culturing ESCs. For example, RGO substrates are free‐standing, electrically conductive, xenogeneic‐free, chemically stable, easily modifiable, and can be used as scaffolding material for further studying cell transplantation and the differentiation of ESCs into electrically sensitive tissues (neural, myocardial, etc.).

It can be seen from FTIR that RGO‐RT possesses many oxygen‐containing groups (hydroxyl groups and carbon–oxygen double bonds), and after the reduction of RGO, the oxygen‐containing groups decrease and the surface contact angle increases, in consistent with the results of other researches.^33^ During the annealing process, the gases (H_2_O, CO_2_, and CO) are generated from the pyrolysis of oxygen groups, which are likely to create some pores and bubble on the RGO substrate.^34^ When the water droplet contacts the calcined substrate, the structure of defects on the surface of the substrate forms a certain interaction force with the water droplet,^35^ and the droplet tends to spread out, resulting in the changes of contact angle on the surface. So, it is speculated that the pores and bubbles of the substrate lead to the deviation of the final contact angle.

It has been reported that the hydrophilicity of the material decreases as the number of oxygen‐containing groups decreases, which is not conducive to the adhesion of stem cells.^28^, ^29^, ^30^ In this work, the number of ESCs adhering to the annealed RGO substrates is significantly reduced, whereas the ESCs on the unannealed substrate not only extend filopodia anchoring to the substrate but also proliferate rapidly, indicating that the surface property (oxygen‐containing groups) of the substrate promotes cell adhesion and proliferation, which is consistent with other studies.^28^, ^29^, ^30^, ^36^ It has been reported that surface property may promote cell adhesion by accelerating the expression of vinculin and fibronectin.^28^, ^29^, ^37^ In this paper, we found that the expression level of adhesive protein E‐cadherin in the annealed RGO substrate is lower than that in the unannealed substrate. Surface property can induce changes in relevant intracellular signaling pathways by modulating the expression of different adhesion proteins.^37^, ^38^ Therefore, we speculate that changes in surface properties caused by different oxygen‐containing groups first changed the expression of some adhesion proteins in ESC, leading to the changes in related intracellular pathways, and ultimately, the ability of ESC to adhere, proliferate, and self‐renew. In this work, CCK‐8 and colony formation assays reveal that RGO‐30 substrate obvious promotes cell survival and self‐renewal compared with RGO‐50 and RGO‐15 substrates. Studies have reported that the geometrical features of the substrate can affect the pluripotency of ESC.^13^, ^14^, ^15^ Although there is no significant difference between RGO‐50, RGO‐30, and RGO‐15 substrates in maintaining ESC pluripotency herein, the reason may be that the pore size interval is too small for ESC. The oxygen‐containing groups of the substrate affect adhesion and proliferation of the cells, and as the oxygen‐containing groups decrease, the expression of pluripotency proteins decreases. Therefore, we speculate that the geometrical features and chemical properties of the RGO substrate cooperate to maintain the pluripotency of ESC.

We also found that ESCs on the RGO substrates highly expressed β‐catenin and E‐cadherin, in which β‐catenin can activate the classic Wnt signaling pathway, which is very important for ESCs maintaining pluripotency. Therefore, we modified the RGO substrate with DKK1 to terminate the classic Wnt signaling pathway. DKK1 can bind to the b‐propeller motif regions of LRP 5/6 and inhibits the binding and activating downstream signals of Wnt ligand, or it may reduce the availability of LRP 5/6 at plasma membrane surfaces.^39^, ^40^ Upon decomposition of the LRP 5/6 receptor complex, the released complex interacts with β‐catenin, leading to its phosphorylation by CK1 and GSK3, which results in the degradation of proteasome and downregulation of Wnt signaling.^41^ After being modified by DKK1, the substrate could no longer maintain the pluripotency of ESCs; furthermore, the intercellular interaction of ESCs on the substrate is hindered, and the expression of E‐cadherin is significantly reduced. Cadherin is essential for cell survival. β‐catenin and cadherin are closely related to cell adhesion and survival mediation. For example, hydrogel not only promotes NPC survival, but also maintains the stemness of NPC via the regulation of cadherin/β‐catenin signaling.^27^ Subsequently, we constructed cell lines that interfered with E‐cadherin and found that the pluripotency of ESCs on the RGO substrates is dependent on E‐cadherin. Through these series of experiments, we have demonstrated that RGO substrates can maintain ESCs by regulating the E‐cadherin/Wnt signaling pathway without sacrificing the pluripotency of ESCs. In studies concerning ESCs cultured on other biomaterial substrates, it has also been reported that the substrate could regulate the protein expression levels of E‐cadherin and β‐catenin,^13^, ^14^, ^16^ which strongly supports our research.

In this work, we have proved through CCK‐8, SEM, and colony formation assays that RGO substrate not only promotes cell survival and self‐renewal, but also maintains ESC pluripotency for a long time. ECM can also promote cell adhesion and proliferation, and maintain ESC pluripotency in the presence of conditioned medium or cytokines.^42^, ^43^, ^44^ RGO and ECM have similar effects on ESC. Therefore, we speculate that RGO substrate may promote high expression of E‐cadherin and β‐catenin by ECM or ECM‐like methods, thereby activating E‐cadherin/Wnt signaling pathway to maintain ESC pluripotency.

Since RGO substrates contain a large number of carboxyl groups, they can be modified via protein manipulation through electrostatic interaction.^45^, ^46^ The protein that binds to the RGO substrate can maintain its structure and bioactivity.^46^, ^47^ In contrast, although protein could bind to graphene via π–π interactions, this binding mode inactivates the protein.^47^ Therefore, RGO substrates can be modified by different signaling pathway proteins to regulate the behavior of stem cells. For example, BMP 2 could be loaded on a GO‐coated titanium substrate via interaction with the carboxyl groups, achieving a sustained release of BMP 2 and promoting osteogenesis of human mesenchymal stem cells.^48^ The method of modifying RGO substrates with a signaling pathway protein not only allows the protein to directly contact the cells, but also prolongs the half‐life of the protein, allowing the protein to exert its function better. In addition, RGOs have good electrical conductivity,^49^, ^50^, ^51^ and we can achieve dual regulation of stem cell behavior through electrical stimulation and signaling pathway protein modification. All of these show the importance of the RGO substrates with regard to our studies concerning the culturing of stem cells and differentiation.

## 4 Conclusion

In summary, we have investigated the effect of RGO substrates possessing different pore sizes and oxygen‐containing group content on the pluripotency of ESCs, in the absence of feeders in vitro. First, we found that the RGO‐30 substrate significantly promotes and sustains the self‐renewal ability of ESCs, and that the ESCs cultured on RGO‐50, RGO‐30, and RGO‐15 substrates possess multilineage differentiation potential. Second, in contrast to the reduced RGO‐300 and RGO‐600 substrates, ESCs on the RGO‐RT substrates not only adhere and proliferate rapidly, but also maintain pluripotency. Finally, RGO substrates not only deepen E‐cadherin‐mediated cell–cell interaction, but also activate the expression of β‐catenin. By modifying the RGO substrate with DKK1 and constructing cell lines that interfered with E‐cadherin, we demonstrated for the first time that the RGO substrates sustain ESC pluripotency by modulating the E‐cadherin/Wnt signaling pathway without sacrificing their self‐renewal ability and multilineage potential. More importantly, graphene and its derivatives possess excellent electrical conductivity and allow for easy surface modification. They are a potential material for future research on the dual regulation of electrical stimulation and signaling pathway protein modification on stem cell differentiation, especially with regard to electrically sensitive tissues.

## 5 Experimental Section

*Culture of Mouse ESCs*: Mouse ESCs were cultured on mitotically inactivated mouse feeder cells in the incubator. The culture medium contained high glucose Dulbecco's modified Eagle medium (DMEM) (Gibco), 15% fetal bovine serum (FBS; Gibco), 1 × GlutaMAX (Gibco), 1 × sodium pyruvate (Gibco), 1 × nonessential amino acids (Gibco), β‐mercaptoethanol (Gibco), and leukemia inhibitory factor (LIF). The culture medium was replaced every day, and mouse ESCs were passaged every 2 days by adding trypsin (Gibco). To remove the feeder cells, the mixture was transferred to a cell culture plate which had been preplated with gelatin (Sigma‐Aldrich) and placed in the incubator for 30 min. Therefore, the feeder cells would be attached to the plate and mouse ESCs were in the supernatant. Then mouse ESCs were inoculated onto feeder cells, gelatin, and RGO substrates, separately.

*Culture of Human ESCs*: Human ESCs were cultured on matrigel (Corning) with mTeSR1 medium (STEMCELL). The matrigel was diluted with DMEM at 1:100 and placed in the incubator for 3 h. Human ESCs were passaged every 5 days and the medium was replaced every day.

The medium was aspirated and washed once in DMEM. The cells were digested in the incubator for about 4 min by adding TrypLE (Gibco), then the TrypLE was aspirated and culture medium was added. The bottom of culture plate was drawn with a pipette to separate cells, and the cell suspension was transferred to the centrifuge tube. Large cell clusters were blown into small clusters with a pipette. Human ESC was generally passed down at a ratio of 1:6–10.

*Synthesis and Characterization of RGO Substrates*: The fabrication process of RGO is illustrated as follows. First, the graphene oxide dispersion was poured into the polytetrafluoroethylene (PTFE) mold and dried for 20–40 h at room temperature to form a film. Then, the obtained graphene oxide film was cut into the desired sizes and immersed in l‐ascorbic acid solution with at an oven temperature of 95 °C over 3 h. The repulsive interaction among GO layers and the cross‐linking between ions and oxygen‐containing groups attains a balance. l‐ascorbic acid solution acts as a reductant and can weakly reduce the graphene oxide films to RGOs. The anisotropic porous RGO substrates were obtained and the samples were freeze‐dried for 12 h. At this time, the substrate could be used for stem cell culturing. Finally, in order to study the effect of different amounts of oxygen‐containing groups present in the substrates on cell behavior and fate, the substrates were thermally annealed before cell seeding under an Ar atmosphere at 300 and 600 °C for 3 h, respectively. The arbitrary‐shaped graphene substrates with different pore sizes were fabricated under the same conditions except using a different concentration of the immersion solution. The pore sizes could be varied over a wide range from 15 to 50 µm. The structure and composition were confirmed by FTIR, Raman spectra, and XRD.

*SEM Imaging of ESCs*: The single cells of mouse ESCs were inoculated onto different substrates and cultured for 4 h in the incubator. Subsequently, they were immersed in 2.5% glutaraldehyde (Alfa Aesar) solution and fixed overnight at 4 °C. The cells were washed three times with water at 4 °C, and gradient dehydrated in 10%, 30%, 50%, 70%, and 90% ethanol and placed at 4 °C for 10 min, respectively. The samples were also dehydrated three times in 100% ethanol, every 30 min. Subsequently, the samples were transferred to isopropanol (Sinopharm Chemical Reagent Co., Ltd), frozen in liquid nitrogen for about 10 min, and immediately freeze‐dried. Finally, the samples were observed under a scanning electron microscope.

*Spontaneous Differentiation*: The spontaneous differentiation medium was composed of high glucose DMEM (Gibco), 15% fetal bovine serum (Gibco), 1 × GlutaMAX (Gibco), 1 × sodium pyruvate (Gibco), 1 × nonessential amino acids (Gibco), and β‐mercaptoethanol (Gibco). After trypsinizing mouse ESCs and removing the feeder cells, mouse ESCs were harvested. The mouse ESCs were resuspended in a spontaneous differentiation medium and transferred to cell culture plate that had been coated with gelatin in advance. The cells were cultured for 4 days, and the differentiation medium was replaced every day. The cells that differentiated for 0, 2, 3, and 4 day(s) were collected respectively for performing qRT‐PCR.

*Retinoic Acid Differentiation*: The mouse ESCs were harvested by trypsinization and removal of feeder cells. Next, the cells in RA (Sigma‐Aldrich) differentiation medium, containing 1 µmol mL^−1^ RA, were cultured in cell culture plate for 5 days. The RA differentiation medium was replaced every day. The cells that differentiated for 0, 3, 4, 5 day(s) separately were collected and qRT‐PCR was performed.

*Vector*: The short hairpin RNAs (shRNAs) targeting E‐cadherin were designed and inserted into pLKO.1 cloning vector to generate shE‐cadherin. The complementary DNA (cDNA) for the E‐cadherin gene was cloned into the Flip, ubiquitin promoter, GFP and WRE vector. The recombinant plasmid was confirmed by DNA sequencing, and the primer sequences used in this research are listed in Table S1 (Supporting Information).

*Modification of RGO Substrate*: Dickkopf‐related protein 1 (DKK1) was purchased from Sino biological. The method of modifying graphene oxide with protein was based on previously published research.^23^ Water and DKK1 (10 µg mL^−1^), diluted with water, were separately added to the RGO‐30 substrates and then the substrates were cooled at 4 °C for 2 h. The water‐modified RGO substrate was used as a control, and the modification was detected by western blotting.

*qRT‐PCR*: The total RNA content of the cells was extracted by RNAiso Plus reagent (Takara, 9109), and the concentration was measured using a Malcom e‐spect fluorophotometer. The total RNA content was reverse transcribed into cDNA using PrimeScript RT Reagent Kit (Takara). qRT‐PCR was carried out on an Agilent Technologies Stratagene Mx3000P using iTaq Universal SYBR Green Supermix (Bio‐rad). Glyceraldehyde‐3‐phosphate dehydrogenase gene was used as the internal reference. All the experiments were repeated three times. The primer sequences employed in this research are listed in Table S1 (Supporting Information).

*Immunofluorescence Staining*: The cells were washed once with phosphate buffer solution (PBS) after aspirating the culture medium. Then they were fixed with 4% paraformaldehyde (PFA) for 20 min at room temperature. 0.2% TritonX‐100 was used to permeate the fixed cells for 8 min. The cells were blocked with 10% FBS for 1 h at room temperature and incubated with primary antibody (anti‐Nanog, Abcam; anti‐Oct4, Abcam; anti‐E‐cadherin, Abcam; anti‐β‐catenin, Merck Millipore) overnight at 4 °C. Subsequently, the cells were exposed to a fluorescent secondary antibody for 3 h at 4 °C, and the cell nuclei were stained with Hoechst 33 342 (Ho.33 342) for 30 min at room temperature. Finally, the samples were observed under a fluorescence microscope.

*Alkaline Phosphatase Assay*: After the mouse ESCs were trypsinized, 600 single cells were added to one well of 6‐well culture plate and cultured for 5 days. The cells were washed once with PBS and fixed with 4% PFA for 1 min. The fixed cells were washed once with PBS, and 1 mL of staining solution (Sigma‐Aldrich) was then added to the cells, heating them at 37 °C for 20 min. Finally, the cells were washed twice with PBS and photographed.

*Western Blotting*: The cells were washed once with PBS and the appropriate amount of sodium dodecyl sulfonate (SDS) buffer was added. Subsequently, the samples were sonicated on ice for 25 s for complete cell lysis. The cell lysates were denatured in a metal bath at 95 °C for 10 min. The same number of samples were separated by SDS–polyacrylamide gel electrophoresis (SDS–PAGE) and then they were transferred to a polyvinylidene fluoride (PVDF) membrane. The PVDF membrane was blocked with 3% bovine serum albumin for 1 h at room temperature and incubated with primary antibody (anti‐E‐cadherin, Cell Signaling Technology; anti‐β‐tubulin, AB clonal) overnight at 4 °C. The membrane was then incubated with the corresponding secondary antibody for 2 h at 4 °C. The blots were imaged through FluorChem E by chemiluminescence with Clarity Western ECL Substrate (Bio‐rad). For the western blotting of DKK1, the aim was to confirm whether RGO could be modified by DKK1, so there was no cell participation in this process. An equal amount of SDS buffer was added to DKK1‐ or water‐modified RGO. After the lysates were denatured in a metal bath at 95 °C, the same number of lysates was separated by SDS–PAGE and transferred to a PVDF membrane. The blots were then blocked, incubated with primary and secondary antibodies.

*Statistical Analysis*: The data were analyzed by GraphPad Prism 5 with two‐tailed Student's *t*‐test (*n* ≥ 3). The standard error of the mean was represented by error bar. ^#^/*, ^##^/**, ^###^/*** stand for *p* < 0.05, *p* < 0.01, and *p* < 0.001, respectively.

## Conflict of Interest

The authors declare no conflict of interest.

## Supporting information

Supplementary
