# Supplementary material for: Structurally Tunable Reduced Graphene Oxide Substrate Maintains Mouse Embryonic Stem Cell Pluripotency
Source: Adv Sci (Weinh). 2019 Apr 17;6(12):1802136. doi: 10.1002/advs.201802136 (PMC6662269; doi:10.1002/advs.201802136)
Supplement: Supplementary file 1 — Supplementary [file ADVS-6-1802136-s001.pdf]

Copyright WILEY-VCH Verlag GmbH & Co. KGaA, 69469 Weinheim, Germany, 2019.

ADVANCED SCIENCE
Open Access

Supporting Information

for *Adv. Sci.,* DOI: 10.1002/advs.201802136

Structurally Tunable Reduced Graphene Oxide Substrate Maintains Mouse Embryonic Stem Cell Pluripotency

Jinping Zhao, Mingliang Tang, Jing Cao, Dan Ye, Xudong Guo, Jiajie Xi, Yi Zhou, Yuchen Xia, Jing Qiao, Renjie Chai, Xiaowei Yang,* and Jiuhong Kang**

Supporting Information

Structurally Tunable Reduced Graphene Oxide Substrate Maintains Mouse Embryonic Stem Cell Pluripotency

Jinping Zhao, Mingliang Tang, Jing Cao, Dan Ye, Xudong Guo, Jiajie Xi, Yi Zhou, Yuchen Xia, Jing Qiao, Renjie Chai, Xiaowei Yang* and Jiuhong Kang**

J. Zhao, Dr. D. Ye, Dr. X. Guo, Dr. J. Xi, Y. Xia, J. Qiao, Prof. J. Kang
Clinical and Translational Research Center of Shanghai First Maternity and Infant Health Hospital, School of Life Science and Technology, Tongji University, Shanghai 200092, China
E-mail: jhkang@tongji.edu.cn

J. Zhao, J. Cao, Y. Zhou, Prof. X. Yang
Institute for Regenerative Medicine, Shanghai East Hospital, School of Materials Science and Engineering, Tongji University, Shanghai 200092, China
E-mail: yangxw@tongji.edu.cn

Prof. M. Tang, Prof. R. Chai
Key Laboratory for Developmental Genes and Human Disease, Ministry of Education, Institute of Life Sciences, Jiangsu Province High-Tech Key Laboratory for Bio-Medical Research, Southeast University, Nanjing 210096, China.
Co-Innovation Center of Neuroregeneration, Nantong University, Nantong 226001, China.
Institute for Stem Cell and Regeneration, Chinese Academy of Science, Beijing, China.
Email: renjiec@seu.edu.cn

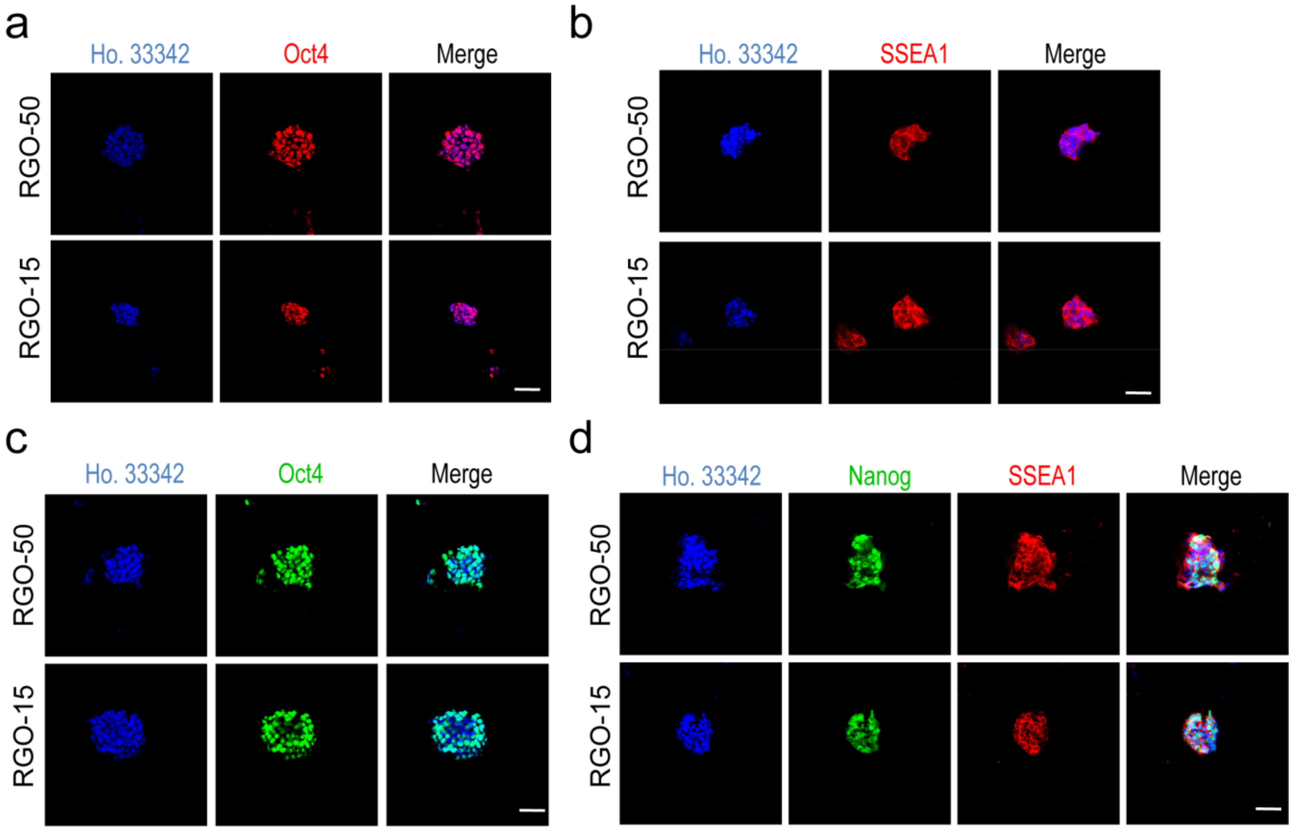

Figure S1. The protein levels of Oct4 (a) and SSEA1 (b) in NR3 cultured on RGO-50 and RGO-15 substrates. Scale bar: 50 µm in (a, b). The immunofluorescent staining of Oct4 (c), Nanog (d) and SSEA1 (d) in 46C cultured on RGO-50 and RGO-15 substrates. Scale bar: 50 µm in (c, d).

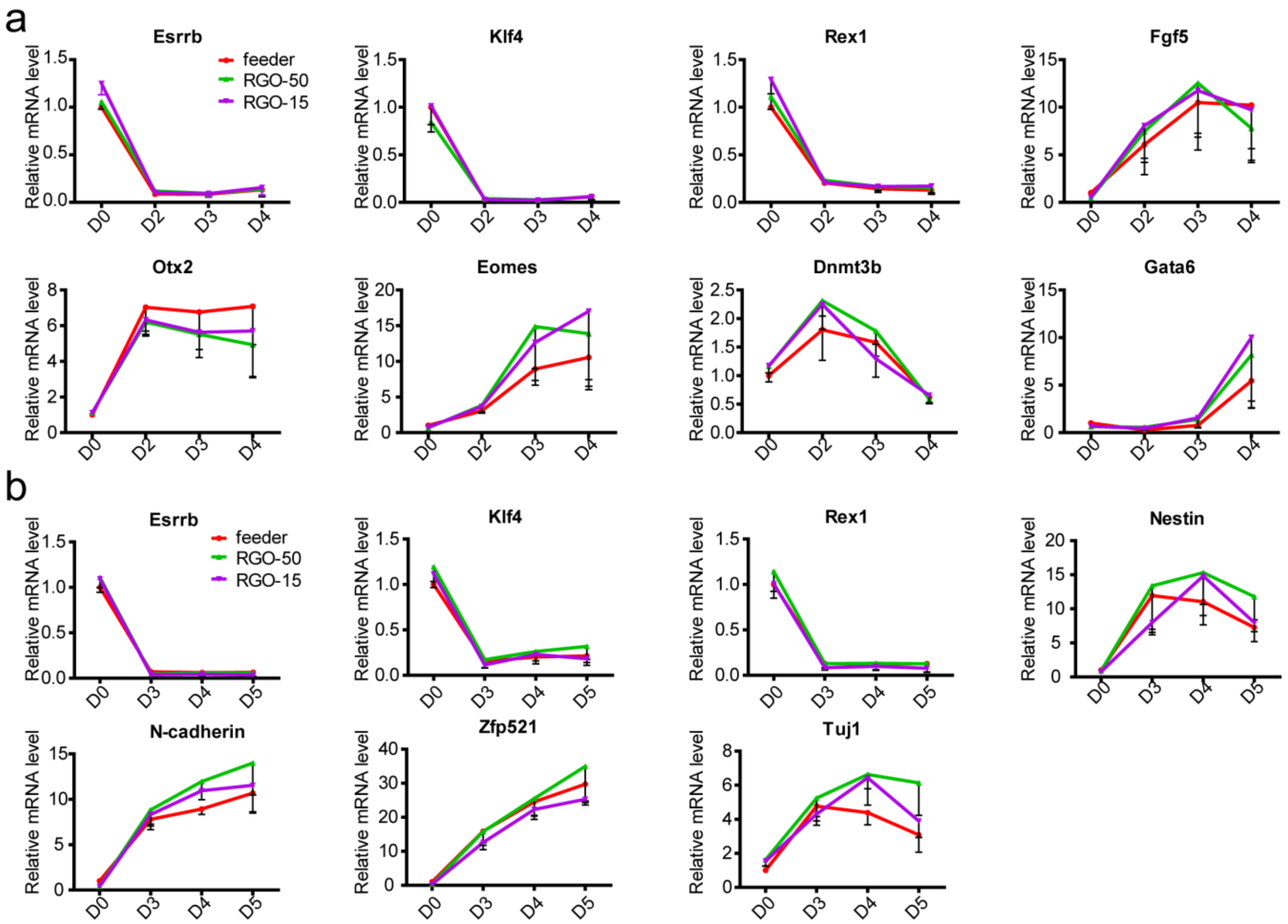

Figure S2. a) qRT-PCR was performed to analyse the relative levels of pluripotency gene and mesendoderm gene during early differentiation in 46C on RGO-50 and RGO-15 substrates. b) The relative expression levels of Esrrb, Klf4, Rex1, Nestin, N-cad, Zfp521 and Tuj1 in 46C cultured on RGO-50 and RGO-15 substrates.

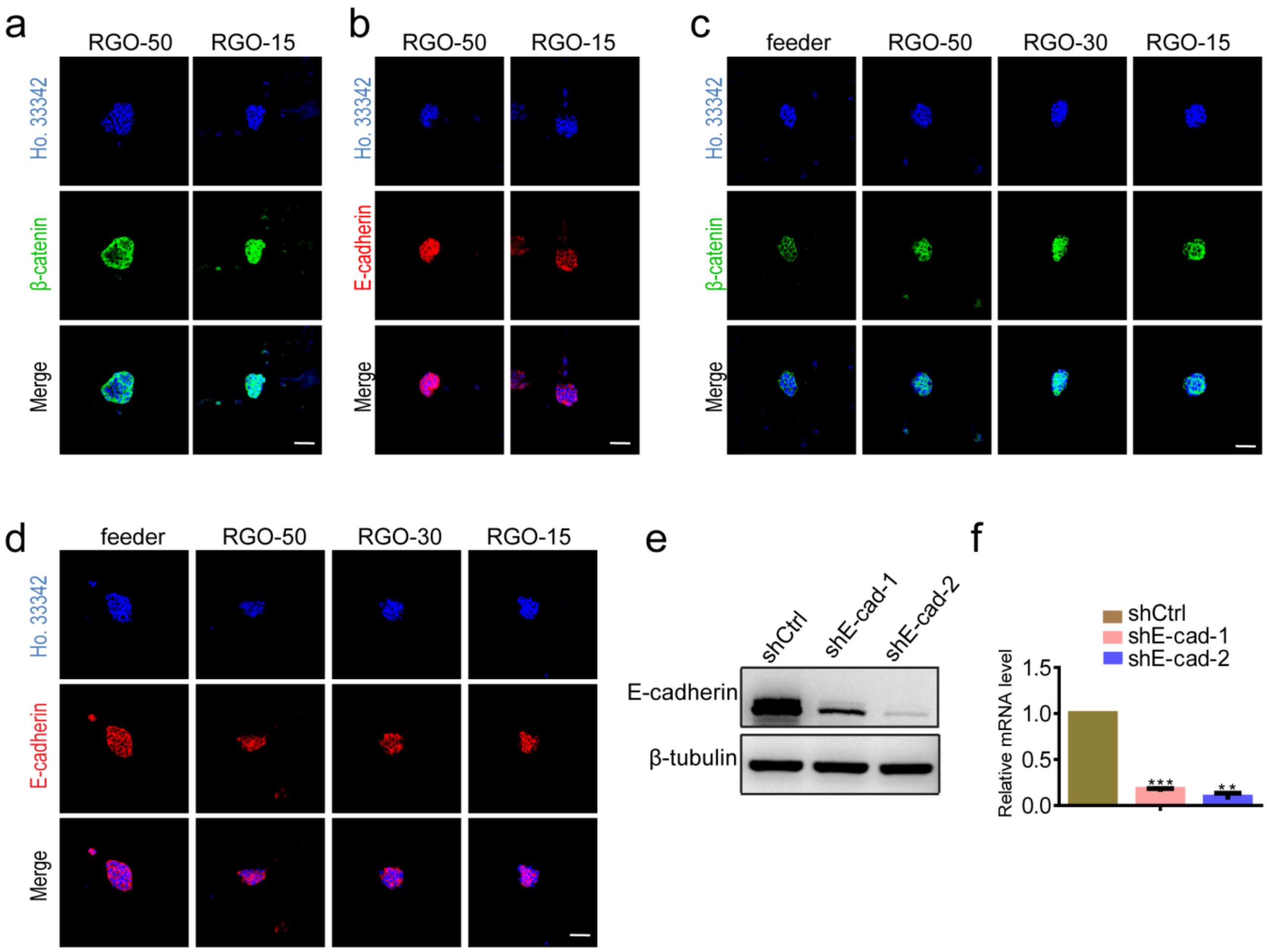

Figure S3. The fluorescence microscope images of β-catenin (a) and E-cadherin (b) in 46C on RGO-50 and RGO-15 substrates. Scale bar: 50 μm. The protein expression levels of β-catenin (c) and E-cadherin (d) of NR3 cultured on feeder, RGO-50, RGO-30 and RGO-15 substrates. Scale bar: 50 μm in (c, d). e) The western blotting of E-cadherin in shCtrl, shE-cad-1 and shE-cad-2 cultured on RGO-30 substrate. f) The expression level of E-cadherin in shE-cad-1 and shE-cad-2 cultured on RGO-30 substrate was confirmed by qRT-PCR. ** $p < 0.01$, *** $p < 0.001$ versus shCtrl group.

Table S1. The list of primer sequences

Application	Gene	Forward primer	Reverse primer
shRNA	shE-cad-1	CCGGGCTGGAATCTTTGTCCATGTACTCGAGTACATGGACAAAGATTCCAGCTTTTTG	AATTCAAAAAGCTGGAATCTTTGTCCATGTACTCGAGTACATGGACAAAGATTCCAGC
shRNA	shE-cad-2	CCGGCCGAGAGAGTTACCCTACATACTCGAGTATGTAGGGTAACTCTCTCGGTTTTTG	AATTCAAAAACCGAGAGAGTTACCCTACATACTCGAGTATGTAGGGTAACTCTCTCGG
qRT-PCR	Nanog	CAGGTGTTTGAGGGTAGCTC	CGGTTCATCATGGTACAGTC
qRT-PCR	Oct4	TCTTTCCACCAGGCCCCCGGCTC	TGCGGGCGGACATGGGGAGATCC
qRT-PCR	Sox2	GATCAGCATGTACCTCCCC	CCCTCCCAATTCCCTTGTATC
qRT-PCR	Klf4	GTGCAGCTTGCAGCAGTAAC	AGCGAGTTGGAAAGGATAAAGTC
qRT-PCR	Esrrb	TGGCAGGCAAGGATGACAGA	TTTACATGAGGGCCGTGGGA
qRT-PCR	Rex1	GGAAGAAATGCTGAAGGTGGAGAC	AGTCCCCATCCCCTTCAATAGC
qRT-PCR	Fgf5	AAAGTCAATGGCTCCCACGAA	GGCACTTGCATGGAGTTTTCC
qRT-PCR	Otx2	CCATGACCTATACTCAGGCTTCAGG	GAAGCTCCATATCCCTGGGTGGAAAG
qRT-PCR	Eomes	CCTGGTGGTGTTTTGTTGTG	TTTAATAGCACCGGGCACTC
qRT-PCR	Dnmt3b	AGCGGGTATGAGGAGTGCAT	GGGAGCATCCTTCGTGTCTG
qRT-PCR	Gata6	GACGGCACCGGTCATTACC	ACAGTTGGCACAGGACAGTCC
qRT-PCR	Nestin	CTGCAGGCCACTGAAAAGTT	GACCCTGCTTCTCCTGCTC
qRT-PCR	Zfp521	GAGCGAAGAGGAGTTTTTGG	AGTTCCAAGGTGGAGGTCAC
qRT-PCR	N-cadherin	TCCTGATATATGCCCAAGACAA	TGACCCAGTCTCTCTTCTGC
qRT-PCR	Tuj1	TAGACCCCAGCGGCAACTAT	GTTCCAGGTTCCAAGTCCACC
qRT-PCR	E-cadherin	CAGCCTTCTTTTCGGAAGACT	GGTAGACAGCTCCCTATGACTG
qRT-PCR	GAPDH	GTGTTCCTACCCCCAATGTGT	ATTGTCATACCAGGAAATGAGCTT
